# Clinical and systems of care factors contributing to individual patient decision-making for early mobilization post-stroke

**DOI:** 10.3389/fstro.2023.1293942

**Published:** 2023-12-07

**Authors:** Venesha Rethnam, Kathryn S. Hayward, Hannah Johns, Lilian B. Carvalho, Leonid Churilov, Julie Bernhardt

**Affiliations:** ^1^The Florey Institute of Neuroscience and Mental Health, Heidelberg, VIC, Australia; ^2^National Health and Medical Research Council (NHMRC) Centre for Research Excellence in Stroke Rehabilitation and Brain Recovery, Melbourne, VIC, Australia; ^3^Melbourne School of Health Sciences, University of Melbourne, Melbourne, VIC, Australia; ^4^Melbourne Medical School, University of Melbourne, Melbourne, VIC, Australia; ^5^Melbourne Brain Centre, Royal Melbourne Hospital, Melbourne, VIC, Australia

**Keywords:** stroke, mobility, early mobilization, acute, recovery, rehabilitation, guidelines

## Abstract

**Introduction:**

Many stroke guidelines recommend against starting intensive out-of-bed activity (mobilization) within 24 h post-stroke. Few guidelines address care after the first 24–48 h, and little information is provided about how early mobilization decisions should be tailored to patients. We aimed to identify clinical and systems of care factors contributing to individual patient decision-making for early mobilization post-stroke.

**Methods:**

Expert stroke clinicians were recruited to participate in an interactive one-on-one session that included an introductory semi-structured interview followed by an assisted data exploration session using an early mobilization data visualization tool.

**Results:**

Thirty expert stroke clinicians with a median (interquartile range) 14 (10–25) years of experience were included. Stroke type and severity, and medical stability were identified as important clinical decision-making factors by the majority of expert stroke clinicians. Inadequate staffing and equipment were frequently indicated as barriers to early mobilization. The perceived characteristics of early mobilization responders were mild or moderate stroke severity, ischemic stroke, partial anterior circulation stroke, younger age, and one or fewer comorbidities. Perceived characteristics of early mobilization non-responders included severe stroke severity, hemorrhagic stroke, total anterior circulation stroke, older age, those with persistent vessel occlusion or high-grade stenosis, hemodynamic instability, multimorbidity and an altered state of consciousness. Some characteristics led to uncertainty amongst interviewees e.g., early mobilization decision-making were moderate stroke severity, older patients, and those with lacunar circulation infarcts.

**Discussion:**

We gained unique, in-depth insights into patient and systems of care factors that contribute to individual patient decision-making related to early mobilization post-stroke. The identified areas would benefit from further empirical research to develop structured decision support for clinicians.

## 1 Introduction

It is well-established that the delivery of care in organized acute stroke units is effective in reducing death and long-term dependency (Langhorne et al., 2020). Early mobilization, defined as sitting out of bed, standing or walking early after stroke, is a component of this care model (Bernhardt et al., 2015). The susceptibility of patient inactivity during hospitalization (Bernhardt et al., 2004; Sheedy et al., 2020) and the increased risk of immobility-related complications such as infections, falls, and thromboembolism leading to poor functional recovery, forms the central theoretical rationale for early mobilization (Langhorne et al., 2000; Askim et al., 2014; AVERT Trial Collaboration Group, 2015; Naito et al., 2020). Equally, some concerns exist about early mobilization including increased risk of falls (Naito et al., 2020), impairment of cerebral blood flow and perfusion due to hemodynamic changes (Anderson et al., 2017; Carvalho et al., 2020), and risk of further bleeding in hemorrhagic strokes (Skarin et al., 2011) or after thrombolysis (Muhl et al., 2014).

Significant changes to clinical practice guidelines after the publication of the single largest early mobilization randomized controlled trial, A Very Early Rehabilitation Trial (AVERT; *n* = 2,104) (AVERT Trial Collaboration Group, 2015), which demonstrated poorer outcomes in the early mobilization group compared to usual care. Many guidelines now recommend against starting (intensive) out-of-bed activity within 24 h post-stroke (Bayley et al., 2017). Few guidelines address care after the first 24–48 h. Of those guidelines that recommend early mobilization within 48 h, they contain little information about how this should be tailored to individual patients (Norwegian Directorate of Health, 2017; Boulanger et al., 2018; NICE, 2019; Powers et al., 2019; Stroke Foundation, 2022).

We currently do not know how clinicians make individualized clinical decisions about which patients should be mobilized, particularly in the absence of clinical trials that are powered to detect subgroup effects. Differences in decision-making have been shown to be influenced by the interpretation and understanding of the evidence, the heterogeneity of stroke factors, and individual clinical judgements of the benefits and harms of early mobilization to different patient subgroups (Sjöholm et al., 2011; Skarin et al., 2011; Bernhardt et al., 2015). It is important to understand the specific clinical and non-clinical factors that influence clinicians to tailor early mobilization decision-making.

We conducted an interactive session with expert clinicians that included an introductory semi-structured interview followed by an assisted data exploration using an early mobilization data visualization tool. The aim was to: (1) investigate important patient and systems of care factors influencing early mobilization decision-making; and (2) identify high-interest patient subgroups, i.e., suspected responders (patients who might benefit from early mobilization), non-responders (patients who might be harmed from early mobilization), and patient subgroups that create uncertainty in early mobilization decision-making.

## 2 Methods

This cross-sectional study is reported in accordance with Strengthening the Reporting of Observational Studies in Epidemiology (STROBE) guidelines (von Elm et al., 2007).

### 2.1 Standard protocol approvals, registrations, and patient consents

Ethics approval was obtained from The University of Melbourne Human Research Ethics Committee (1851680.1). Written informed consent was obtained from all participants involved in the study.

### 2.2 Study participants and recruitment

Expert sampling (Etikan et al., 2016) was used to recruit a representative sample of experienced stroke clinicians. The role of identified individuals was to provide an in-depth understanding of the clinical and non-clinical factors that influence early mobilization decision-making. Our sample targeted senior Australian stroke physicians, physiotherapists and nurses/nurse practitioners with a high level of expertise in the delivery of acute stroke care and early mobilization practices (senior position, consistent with >6 years stroke-specific experience). Clinicians identified as appropriate for participation according to this sampling strategy were invited to participate via email and were recruited between June 2019 to March 2020.

### 2.3 Introductory semi-structured interview

The interviews were semi-structured, and questions were designed to elicit the desired topics of interest: important clinical and non-clinical factors for effective decision-making and high-interest patient subgroups (Appendix 1). Participants were encouraged to provide more detailed responses using probing techniques and prompts. All participants were interviewed either in person or via videoconference. Demographic data on participants were collected using a brief questionnaire. A section of the introductory interview had an independent aim to investigate the utility and limitations of current early mobilization clinical practice guidelines. The results of this investigation are reported elsewhere (Rethnam et al., 2021).

### 2.4 Assisted data exploration

The AVERT Atlas is an interactive data visualization tool (Appendix 2) for understanding and investigating complex clinical trial data from the 2,104 patients included in AVERT. It was developed to be run using the statistical software R (R Core Team, 2020), and was built using the Shiny (Chang et al., 2020) and ggplot2 (Wilkinson, 2011) packages. The AVERT Atlas allows a user to select a group-level combination of different patient, stroke, and dose variables as well as the outcome on the modified Rankin Scale (mRS) collected in AVERT. Based on the subgroup selection, the participant could visualize the within-subgroup distribution of the mRS, represented by a segmented bar chart. The investigator (VR) assisted the navigation of the tool if this was desired by the clinical experts. To achieve the aim of this study, the tool was used to investigate influential clinical factors that represent high-interest patient subgroups that represent perceived characteristics of patients who might benefit from early mobilization (responders), patients who might be harmed from early mobilization (non-responders), and patient subgroups that create uncertainty in decision-making. Participants were given a list of all available variables and subsequently asked three questions:

- Which variables define the patient group of highest interest to you?

Prompt: This could be a group that you believe to be a responders or non-responders or a group that you are uncertain about.

- Why are you interested in this group of patients?
- What response to early mobilization do you intuitively anticipate for this group, and why?

### 2.5 Coding and analysis

The interviews were audio-recorded and transcribed verbatim using a paid transcription program (Amberscript) by one author (VR) to facilitate the analysis of data. A deductive and directed thematic content approach (Hsieh and Shannon, 2005) was used based on the main outcomes for the study: (1) the frequency distribution of individual decision-making factors being nominated by stroke clinicians and (2) the frequency distribution of patient and stroke variables used to define patient subgroups of interest. One author (VR) read all transcripts to make initial analytical observations about the data. Transcripts were imported into QSR International NVivo 9 to code and analyse data using a deductive approach. As per the directed thematic content approach, predetermined codes were formed based on the set of a priori and predefined interview questions (Potter and Levine-Donnerstein, 1999). The strategy for coding involved reading through each transcript and conducting line-by-line coding using the predetermined codes. Subcodes were determined during this process with subsequent analysis. The data that could not be initially coded were identified and analyzed later to determine if they represented a new category or a subcategory of an existing code.

A second author (KH) double-coded 25% of all transcript across all coding themes. Differences which occurred were resolved by consensus. The rationale for only double-coding 25% of transcripts was due to the deductive and directed thematic content approach that was utilized. In this top-down process approach, many of the codes were predetermined and aligned directly with the interview questions. Ongoing discussions between the two reviewers established trustworthiness and credibility to clarify the interpretation of the data. All subcodes were discussed between VR and KH to determine overlap or divergence in sub-themes within the broad predetermined codes. VR and KH also randomly selected codes in NVivo to ensure the quotes from the transcripts accurately reflected the theme it was coded to. Descriptive statistics with frequencies and proportions were produced and reported using Stata (version 14.2; StataCorp LP, College Station, TX, USA). Representative verbatim quotes were selected for presentation.

## 3 Results

Thirty clinical stroke experts participated in this study: 11 physicians, 11 physiotherapists and eight nurses (Table 1). On average, clinicians had been practicing in a stroke context for 14 years (IQR 10–25), and 50% highly rated their current level of knowledge on early mobilization evidence.

**Table 1:** Demographic characteristics of expert stroke clinicians.

**Variables**	**No. (%)**
**Occupation**	
Physician	11 (37%)
Physiotherapist	11 (37%)
Nurse	8 (26%)
**Highest level of education**	
Ph.D.	14 (47%)
Clinical doctorate	1 (3%)
Masters (clinical)	5 (17%)
Bachelor's degree	7 (23%)
Graduate diploma	3 (10%)
**Currently working in a stroke service**	
Yes	26 (87%)
No	4 (13%)
**Primary stroke environment**	
Acute stroke unit	22 (74%)
Inpatient rehabilitation	3 (10%)
Outpatient rehabilitation	1 (3%)
Research institute	4 (13%)
**Level of knowledge on early mobilization (self reported)**	
High, well informed about evidence	15 (50%)
Average, up to date with evidence	15 (50%)
Low, not up to date with evidence	0 (0%)
Number of years practicing in a stroke context, median (IQR)	14 (10–25)

IQR, interquartile range.

### 3.1 Introductory interview: clinical factors

Table 2 displays the frequency distribution of clinical factors being nominated by stroke clinicians as important for decision-making. More than 80% of the sample considered stroke severity, medical stability (e.g., hemodynamic stability, cardiovascular stability, risk of complication, respiratory stability and febrile) and type of stroke to be the most important factors contributing to decision-making about early mobilization practices. Around 50% of the sample considered age, delivery of thrombolysis and/or endovascular clot retrieval, and pre-morbid comorbidities to be important clinical factors.

**Table 2:** List of clinical factors considered important to early mobilization decision-making.

**Clinical factors**	***n* (%)**	**Example quotation**
Stroke severity	27 (90%)	PA24 (Physio^*^): “I think the first things that you see as a clinician are their stroke factors. So, what was their stroke severity? So, what was their NIHSS?… I actually think that's probably a big driver…”
Medical stability	25 (83%)	PA2 (Doctor): Also, to consider how sick they are if they have an infection and all these things come into play. Do they have complications? That probably contributes to this mix of “How stable are they?” PA17 (Physio): “But the things I'll be looking at would be medical stability. So, you know, appropriate blood pressure, heart rate, all those sorts of things.”
Hemodynamic	24 (80%)	
Cardiovascular	13 (43%)	
Risk of complications	10 (33%)	
Respiratory	6 (20%)	
Febrile	4 (13%)	
Type of stroke	24 (80%)	PA5 (Nurse): “In terms of stroke classification, I think that is probably important. So if I had on my list a significant penumbra or presence of large vessel occlusion and that's within the first, you know, I'm going to say eight hours after stroke onset, I'd be very cautious about moving those patients around too much including sitting in bed.” PA6 (Doctor): “With haemorrhages, intuitively, you have to wait until they settle down. Mobilizing them, I think I'd be quite gentle”
Functional ability	18 (60%)	PA25 (Doctor): “They [densely hemiplegic patients] won't be able to mobilise early because they need a lot of assistance and then you judge based on the functional outcome of the patient.” PA28 (Doctor): “If I walk in and they are walking around the stroke unit saying, ‘Can I go home?'. That's a determinant.”
Age	16 (53%)	PA18 (Physio): “Potentially elderly patients that didn't have a good baseline. So then on the flipside of that, there are those patients that had a really good baseline that show good potential.”
Thrombolysis and/or endovascular clot retrieval	16 (53%)	PA30 (Doctor): “Post procedural ECR or thrombolysis, I would not be pushing it at all. I'd just wait and see to make sure the groin is okay.” PA14 (Nurse): “I guess if someone's had thrombolysis, part of our protocol would be to keep them resting in bed for about 24 hours.”
Premorbid comorbidities	15 (50%)	PA26 (Physio): “If they've got loads of comorbidities, you know they're a bit more fragile usually. Premorbid Rankin - that's certainly useful.” PA2 (Doctor): “Their premorbid capabilities in terms of moving and ambulating and age probably play a little bit of a factor in terms of general fitness…”
Premorbid mRS	6 (20%)	
Fitness level	5 (17%)	
Consciousness	11 (37%)	PA13 (Nurse): “The patients…that I feel confident in leaving in bed would be the really altered conscious state patients”
Alertness/Delirium	11 (37%)	PA3 (Physio): “I give therapy, but tiredness or alertness or comfortability, neglect, engagement etc, are the factors that dictate how the intervention is given.” PA23 (Doctor): “I guess also other patient factors unrelated to stroke such as if they were very delirious, I'd sort of say I want to make sure that they're not a high falls risk”
Imaging	8 (27%)	PA2 (Doctor): “Now we also have a little bit more information about the anatomy and pathophysiology, and at the same time there has been a secular trend where more and more people have advanced imaging. So, we also have more information about what vessels are open or which vessels are still blocked. I say be careful when they're still blocked vs. when they are open. Although, I must say it has not been really formalized as a ‘no, don't do it'.”
Persistent occlusion	7 (23%)	
Stenosis	4 (13%)	
Penumbra	3 (10%)	
Frailty	7 (23%)	PA19 (Doctor): “In terms of spending a couple of days in bed, the damage that that might do is probably greater for people who are frail and elderly with borderline mobility to start with.”
Falls risk	6 (20%)	PA23 (Doctor): “I'd sort of say I want to make sure that they're not a high falls risk or other medical issues that might necessitate them staying in bed”
Patients' feeling	6 (20%)	PA29 (Nurse): “The other thing, it's what the patient wants often trumps everything else at times.”
Cognition	5 (17%)	PA25 (Doctor): “Some patients have cognitive impairment or dysphasia so they can't follow commands. Hence why they are not great for mobilization.”
Communication	5 (17%)	PA27 (Nurse): “Whether they had language or communication impairment.
Neglect	3 (10%)	PA11 (Nurse): “It's that moderate [NIHSS] section that I think we [early mobilization] have the biggest impact on because…neglect is a big factor…”
Fatigue	2 (7%)	PA17 (Physio): “I would start intervening, but I would very much grade it depending on… their level of fatigue”

^*^Physio, physiotherapist.

### 3.2 Influential non-clinical factors

Table 3 displays important non-clinical factors that influence early mobilization decision-making. The importance of resources such as adequate staffing, staff experience and the availability of equipment in enabling early mobilization practices was also apparent. The stroke experts expressed that a lack of resources are barriers to implementing early mobilization practices.

**Table 3:** List of non-clinical factors influencing early mobilization decision-making.

**Non-clinical factors**	***n* (%)**	**Example quotation**
Staffing	16 (53%)	PA26 (Physio): “Generally… you'd need two people. A minimum of two people. So, if you can't get that extra pair of hands and it's not going to be safe, you don't do it…”
Equipment	9 (30%)	PA19 (Physio): “This is problematic. I'm getting better at this as you get more senior or working longer in an area, but if you don't have access to equipment or interested staff to support you, then it takes a lot of time and a lot of energy.”
Level of education/Expertise of staff	4 (13%)	PA7 (Physio): “I think it's this there's a discrepancy between that decision-making and whether you get somebody up based on how experienced you are, how comfortable are you moving the actual physical stroke patient”
Hospital appointments	3 (10%)	PA22 (Physio): “So is there a lot of activity happening with this person, in which case, you wouldn't even be thinking about it. You'd just be waiting to see what's going on.”
Organization of care	1 (3%)	PA20 (Doctor): “…the most important thing is where are they. Are they at a stroke unit or not stroke unit? Do they have a team around them which is geared towards thinking about stroke care in an organized way?”

### 3.3 High interest patient subgroups

**Figure 1:**
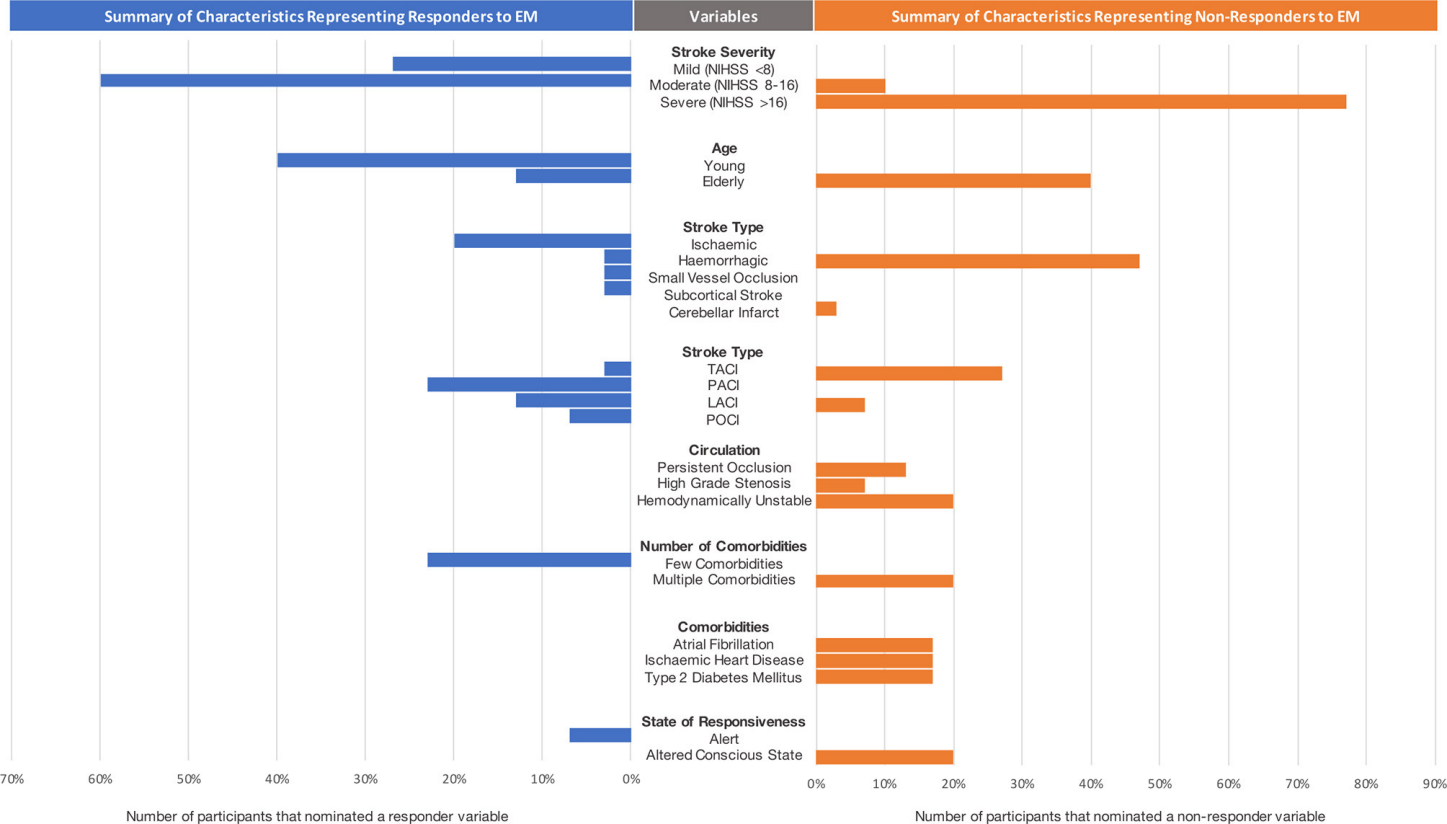
Summary of characteristics representing responders and non-responders to EM decision-making. Note that small vessel occlusion, subcortical stroke and cerebellar infarct were considered as subtypes of ischemic strokes. NIHSS, National Institute of Stroke Scale; TACI, total anterior circulation ischemic stroke; LACI, lacunar ischemic stroke; POCI, posterior circulation stroke.

Figure 1 demonstrates the distribution of selected variables by expert clinicians that represent high interest patient subgroups (responders or non-responders to early mobilization). During the interactive session with stroke experts, the following variables were nominated as characteristics of responders to early mobilization practices: patients with moderate stroke severity (NIHSS 8–16), who are younger (< 65 years old), had an ischemic stroke (particularly small vessel occlusion and subcortical stroke), few comorbidities (0–1) and an alert state of responsiveness.

The variables that were characteristic of non-responders to early mobilization practices included: severe stroke severity (NIHSS > 16), older age, hemorrhagic stroke, total anterior circulation ischemic strokes, persistent occlusion or high-grade stenosis, hemodynamic instability, many comorbidities (e.g., atrial fibrillation, ischemic heart disease and type 2 diabetes mellitus) and altered state of consciousness.

There were several variables that were contradictory in participant responses, i.e., some clinicians identified them as responder characteristics and others as non-responder characteristics, highlighting uncertainty in these subgroups. These variables included people with moderate stroke severity, elderly patients, and those with lacunar infarcts.

## 4 Discussion

The findings from this cross-sectional study provide in-depth insights of key patient and systems of care factors, which are likely to contribute to individualized expert decision-making for early mobilization post-stroke. The novel interactive data visualization session demonstrated the perceived characteristics of suspected responders, non-responders, and patient subgroups that create uncertainty in decision-making for early mobilization post-stroke.

The top three clinical factors that influence early mobilization decision-making were stroke severity, stroke type and medical stability. The importance of stroke severity was reiterated in the interactive data visualization session where many clinicians perceived patients with mild or moderate strokes to be responders, and patients with severe strokes to be non-responders to early mobilization. This finding may reflect the evidence from AVERT, which demonstrated an association (albeit non-significant) between early mobilization and poorer functional outcome in patients with severe stroke (AVERT Trial Collaboration Group, 2015; Kennedy et al., 2021). In contrast, a recent retrospective observational study in Japan in patient with severe stroke found early mobilization was significantly associated with a lower risk of total complication rate of immobility, incidence of pneumonia, and incidence of pressure sore without increasing falls (Naito et al., 2020). People with severe stroke are more likely to develop acute medical complications (particularly immobility-related complications) in the initial hospitalization phase post-stroke resulting in poor functional recovery and death (Indredavik et al., 2008; Boone et al., 2012; Johnson et al., 2019). However, the question regarding the benefits vs. harms of early mobilization in patients with severe stroke remains. This highlights an important area for decision support and guidance for clinicians. Future empirical investigations need to explore this area of uncertainty to help current decision-making processes.

Our findings also demonstrated that stroke type influenced decision-making i.e., patients with ischemic vs. hemorrhagic strokes (Kennedy et al., 2021). Clinicians perceived patients with hemorrhagic stroke to be a subgroup that might respond unfavorably to early mobilization. However, it was interesting that a small sample of clinicians saw some benefit of early mobilization in this subgroup, which highlights the heterogeneity of evidence and opinions. Prior to the publication of AVERT, and during a time with limited decision-support, many stroke clinicians believed patients with hemorrhagic strokes should stay in bed longer than those with ischemic strokes (Skarin et al., 2011). There is some evidence that suggest early mobilization within 24 h might be detrimental for this subgroup (AVERT Trial Collaboration Group, 2015), while other studies suggest that delaying treatment may be harmful (Capo-Lugo et al., 2020; Yen et al., 2020). Exploratory subgroup analyses from AVERT indicated there was a greater odds of death within 14 days post-stroke in those with intracerebral hemorrhage stroke who were treated with early mobilization (started < 24 h post-stroke) compared to usual care (Bernhardt et al., 2020). Still, a recent randomized controlled trial investigating early mobilization within 24–72 h post-stroke in mild to moderate hemorrhagic patients found a significant improvement in early functional independence and shorter length of stay compared to standard early rehabilitation (Yen et al., 2020). Similarly, findings from an ongoing observational cohort study demonstrated that each additional day between admission and initiation of acute rehabilitation therapy (median 3 days) was significantly associated with increased odds of poor outcome (mRS 4–6) at 30 days and at 90 days post hemorrhagic stroke (Capo-Lugo et al., 2020). Perhaps the combination of stroke severity and stroke type factors may result in different responses to early mobilization. Since there is no guidance or specific recommendations after the 24-h period post-stroke for patients with hemorrhagic strokes (Bayley et al., 2017), variability in the delivery of care due to differences in the interpretation of harms vs. benefits is to be expected, and this was demonstrated in our findings.

Finally, medical stability (e.g., hemodynamic stability, cardiovascular stability and risk of complications) was also considered an important factor contributing to decision-making about early mobilization practices. Establishing physiological safety criteria, including systolic blood pressure, heart rate, and consciousness to guide the initiation and progress of early mobilization has been recognized as an important undertaking (Bernhardt et al., 2015). Our findings confirm the need for such criteria.

Age and premorbid comorbidities were, to a lesser extent, considered important factors in early mobilization decision-making. Clinicians perceived patients who are elderly and with many comorbidities to be non-responders and those younger than 65 years with few comorbidities to be responders. This may reflect an understanding of the complex interplay of both factors such that an elderly patient with few morbidities is perceived to have the capacity to respond more favorably to early mobilization than an elderly person with many comorbidities. This was similar to findings in an audit of 300 acute stroke patient records which found patients < 65 years were significantly more likely to receive early mobilization than older patients (Luker et al., 2011). The audit also found quality of care provided by allied health professionals was significantly associated with premorbid independence and comorbidities. It is also likely that patients with significant comorbidity are not represented in clinical trials of early mobilization (likely excluded), creating even greater uncertainty because of the limited evidence base. In summary, the level of comorbid burden of a patient may be an important factor that needs to be considered when assessing the potential benefit or risk of early mobilization for a patient post-stroke.

Around half of the sample considered the delivery of thrombolysis or endovascular clot retrieval to impact early mobilization decision-making. The reasons for these included concerns about groin puncture post endovascular clot retrieval, and increased risk of falls and symptomatic intracerebral hemorrhage post thrombolysis. A study of 54 clinicians using hypothetical vignettes revealed that neurological decline, neurological decline with symptomatic intracerebral hemorrhage, infection of uncertain cause, severe chest infection, severe stroke, drowsiness, and confusion significantly influenced decisions to mobilize thrombolysed patients early post-stroke (Ha et al., 2013). In the absence of decision support or guidance, a tendency to take a conservative approach exist (Ho et al., 2018) in the context of thrombolysis or thrombectomy however, there is no evidence to support this approach (Arnold et al., 2015; Tubergen et al., 2019; Silver et al., 2020, 2023).

Some important systems of care barriers were identified that warrant consideration. This included adequate staffing and the availability of equipment to enable early mobilization practices. This supports a previously published study which identified poor teamwork, inadequate staffing, various organizational barriers, staff attitudes and beliefs, and patient-related barriers to implementing the AVERT early mobilization intervention (Luker et al., 2016). These are important bottlenecks in the delivery of best care and need to be considered when developing decision-support tools such as clinical practice guidelines so that early mobilization is implementable and applicable to the local setting. It may also justify allocation of healthcare resources to ensure best care is delivered and optimal patient outcomes are achieved.

The strength of this study relates to the use of an interactive data visualization tool to observe the expert stroke clinicians' interaction with a large dataset. The tool provided a structured method to explore complex stroke factors. This was advantageous in understanding specific clinical factors, and to elicit high-interest subgroups that require a greater level of clinical judgment in their decision-making process. It must be noted that all participants were repeatedly reminded that that dataset was not powered to look at subgroup effects and therefore, any effects of early mobilization in the subgroups they selected should not influence their current decision-making. Nonetheless, the use of the tool allowed the transition from aggregate data to individual patient data and providing rich information about important decision-making factors.

There are also limitations. Firstly, the expert stroke clinicians in this study were from Australian metropolitan hospitals. As a result, our findings are naturally within the scope of this demographic and may not represent the opinions of clinicians and stroke experts globally, or in regional centers of Australia. However, the multidisciplinary sample of clinical (physicians, physiotherapists, and nurses/nurse practitioners) experts in acute stroke provided rich detail about early mobilization decision-making. Secondly, other allied health professionals who may play an active role in early mobilization were not included. Early work in developing early mobilization trial interventions identified physiotherapists and nurses as the main drivers of early mobilization practices (Bernhardt et al., 2004). Physicians also play an important role in determining whether patients are medically fit to commence early mobilization in cases of uncertainty. We therefore deliberately selected physicians, physiotherapists and nurses for these studies. Future exploration of important early mobilization decision-making factors, systems of care bottlenecks, and perceived responders and non-responders to early mobilization in more geographically and clinically diverse samples may validate the findings from this study. Another limitation is that during the interviews we did not ask clinicians to report the reasons why they indicated specific factors to influence their decisions to mobilize or not stroke patients early post stroke. Knowing the potential motives behind the mentioned factors would have been helpful to understand their decisions. Also, we did not ask clinicians to rank the variables influencing the decision-making process. Since the co-occurrence of multiple variables may have an impact on decision-making, knowing how specific individual factors act on these decisions would be key. These factors should be considered when designing futures studies in this field.

As expected, many clinical and systems of care factors play a fundamental role in decision-making at an individual patient-level. There are some patient and stroke characteristics that determine whether a patient is mobilized early or not, which are not necessarily informed by evidence. We need to be careful that inconclusive and inconsistent early mobilization findings do not instill a sense of complacency or confidence. Nonetheless, these factors have identified important future research areas, and potentially worthwhile patient subgroups that need to be substantiated by empirical investigations to better support clinical decision-making.

## Data availability statement

The raw data supporting the conclusions of this article will be made available by the authors, without undue reservation.

## Ethics statement

The studies involving humans were approved by the University of Melbourne Human Research Ethics Committee (1851680.1). The studies were conducted in accordance with the local legislation and institutional requirements. The participants provided their written informed consent to participate in this study.

## Author contributions

VR: Conceptualization, Data curation, Formal analysis, Investigation, Methodology, Project administration, Writing – original draft. KH: Conceptualization, Formal analysis, Investigation, Methodology, Writing – review & editing. HJ: Formal analysis, Investigation, Writing – review & editing. LBC: Resources, Writing – review & editing. LC: Conceptualization, Formal analysis, Investigation, Methodology, Supervision, Writing – review & editing. JB: Conceptualization, Formal analysis, Investigation, Methodology, Supervision, Writing – review & editing.
