# Supplementary material for: Clinical and systems of care factors contributing to individual patient decision-making for early mobilization post-stroke
Source: Front Stroke. 2023 Dec 7;2:1293942. doi: 10.3389/fstro.2023.1293942 (PMC12802755; doi:10.3389/fstro.2023.1293942)
Supplement: Supplementary file 1 [file Data_Sheet_1.docx]

Appendix 1

Interview Questions on Early Mobilisation Decision-Making

**Introductory Interview Questions**

1. Can you talk me through your decision-making process when thinking about mobilising a person early after their stroke?
2. When thinking about mobilising a patient early after stroke, what clinical factors or resources do you rely on for effective decision-making?
3. What non-clinical factors or resources do you rely on for effective decision-making?
4. Who do you think are responders, and why?
5. Who do you think are non-responders, and why?

**Interactive Session Questions:**

1. Which variables define the patient group of highest interest to you?
   1. Prompt: This could be a group that you believe to be a responders or non-responders or a group that you are uncertain about.
2. Why are you interested in this group of patients?
3. What outcome do you intuitively anticipate for this group and why?

**Appendix 2**


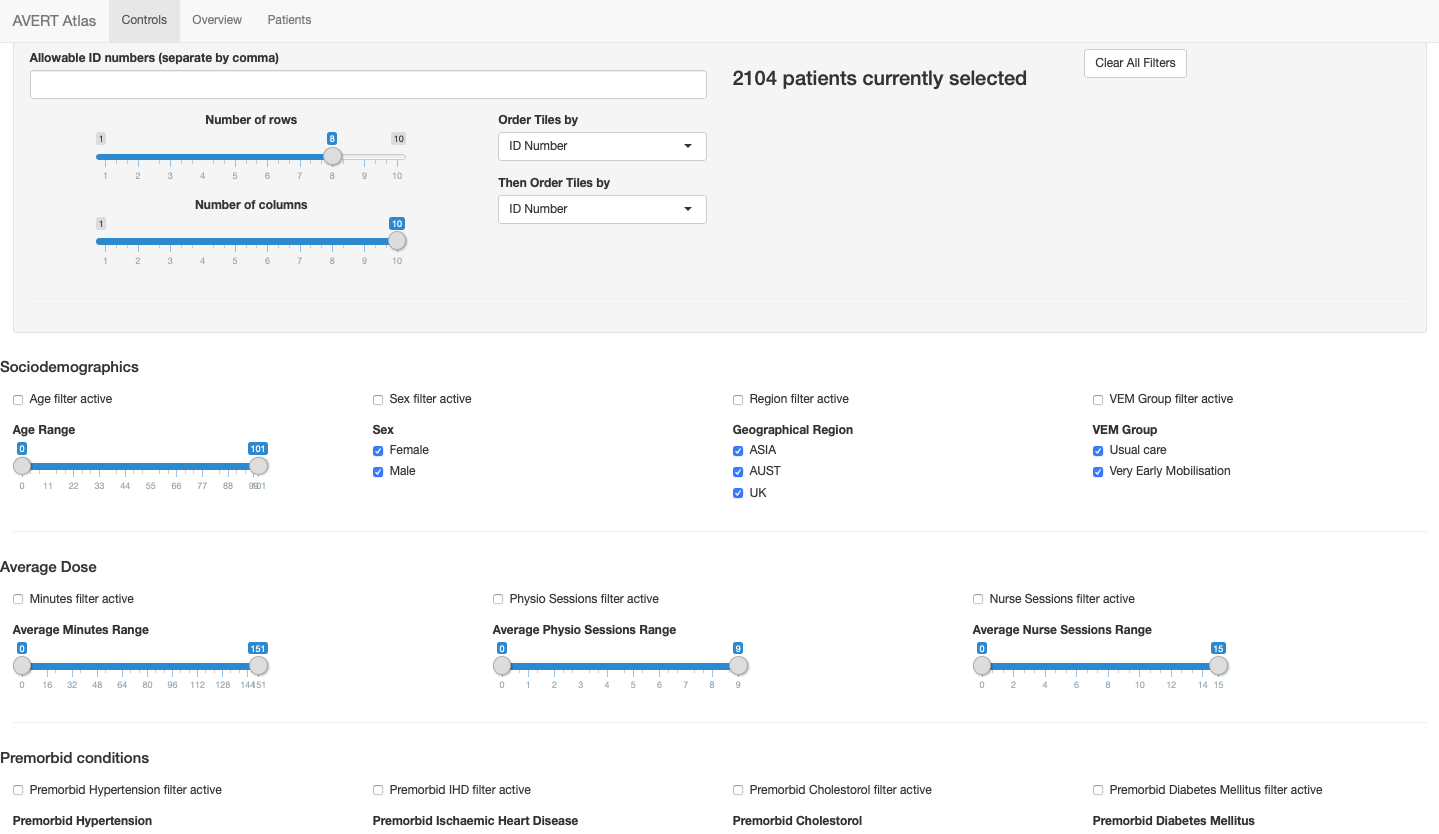


Figure 1. AVERT Atlas *Controls* interface. The figure displays the variables collected in AVERT (A Very Early Rehabilitation Trial) that can be controlled and manipulated by the end user (expert stroke clinician).


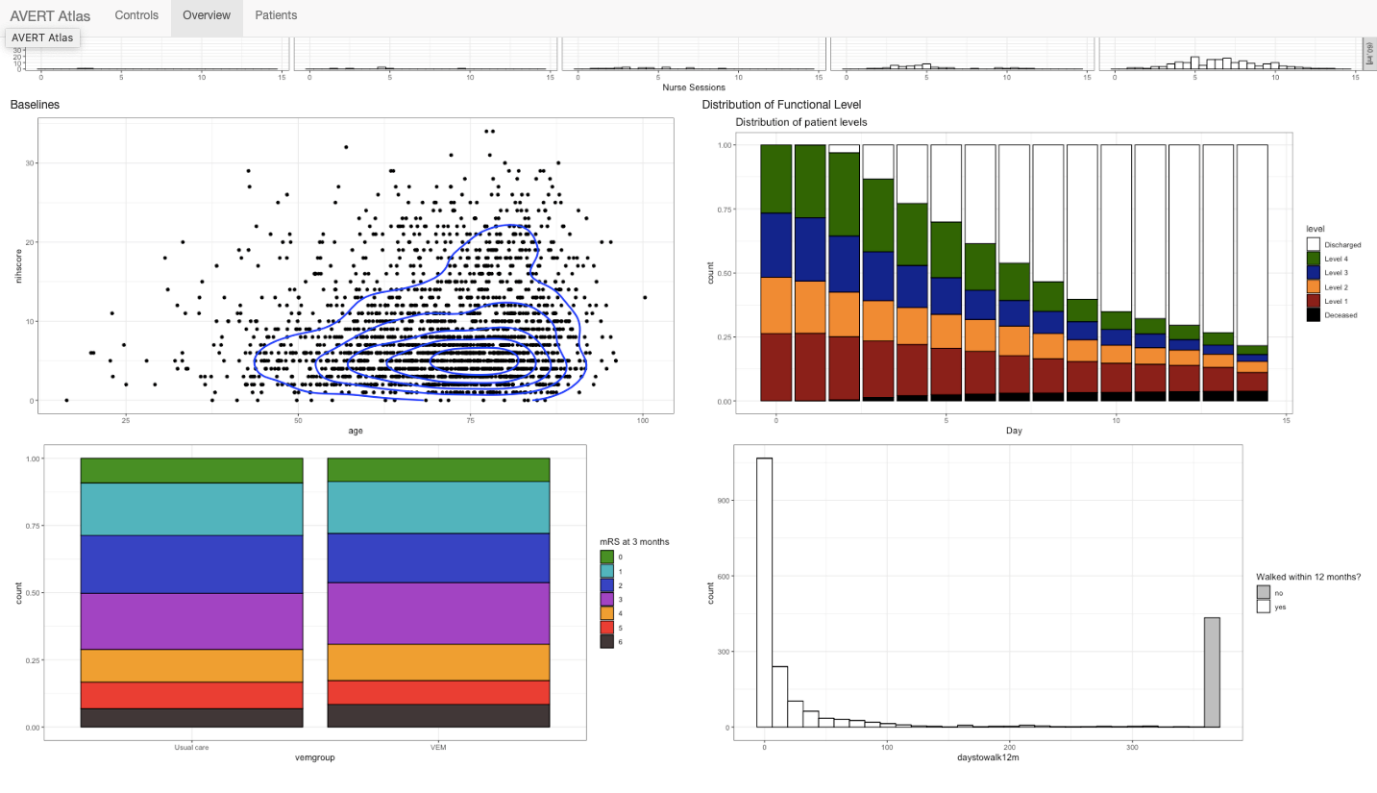


Figure 2. AVERT Atlas *Overview* interface. The figure displays the graphical output of aggregated outcomes based on the selection of variables by the end user (expert stroke clinician).


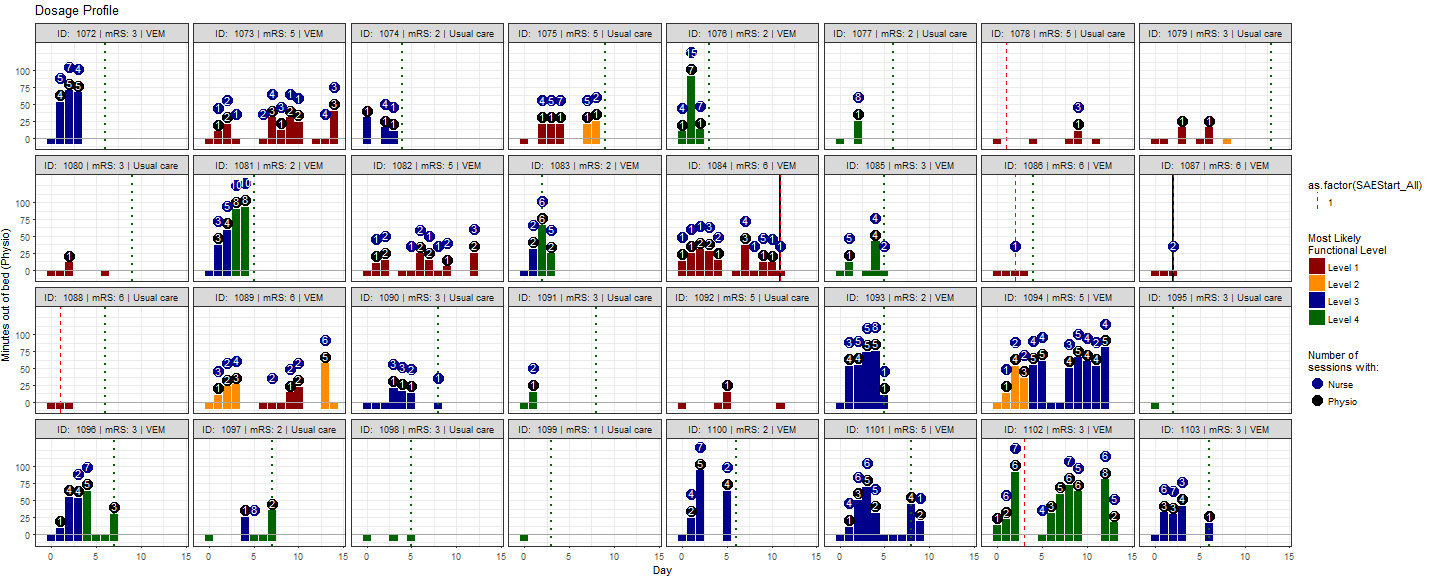


Figure 3. AVERT Atlas *Patients* interface. The figure displays the graphical output of individual patient data based on the selection of variables by the end user (expert stroke clinician). Each tile displays the number of minutes and sessions of out-of-bed activity received by a single participant in AVERT (A Very Early Rehabilitation Trial) each day until discharge or 14 days post-stroke. The colour of each bar graph represents the functional level of the patient
